# The First Mitochondrial Genome for the Superfamily Hagloidea and Implications for Its Systematic Status in Ensifera

**DOI:** 10.1371/journal.pone.0086027

**Published:** 2014-01-21

**Authors:** Zhijun Zhou, Fuming Shi, Ling Zhao

**Affiliations:** 1 College of Life Sciences, Hebei University, Baoding, Hebei Province, China; 2 College of Life Science and Biotechnology, Mianyang Normal University, Mianyang, Sichuan Province, China; University of Houston, United States of America

## Abstract

Hagloidea Handlirsch, 1906 was an ancient group of Ensifera, that was much more diverse in the past extending at least into the Triassic, apparently diminishing in diversity through the Cretaceous, and now only represented by a few extant species. In this paper, we report the complete mitochondrial genome (mitogenome) of *Tarragoilus diuturnus* Gorochov, 2001, representing the first mitogenome of the superfamily Hagloidea. The size of the entire mitogenome of *T. diuturnus* is 16144 bp, containing 13 protein-coding genes (PCGs), 2 ribosomal RNA (rRNA) genes, 22 transfer RNA (tRNA) genes and one control region. The order and orientation of the gene arrangement pattern is identical to that of *D. yakuba* and most ensiferans species. A phylogenomic analysis was carried out based on the concatenated dataset of 13 PCGs and 2 rRNA genes from mitogenome sequences of 15 ensiferan species, comprising four superfamilies Grylloidea, Tettigonioidae, Rhaphidophoroidea and Hagloidea. Both maximum likelihood and Bayesian inference analyses strongly support Hagloidea *T. diuturnus* and Rhaphidophoroidea *Troglophilus neglectus* as forming a monophyletic group, sister to the Tettigonioidea. The relationships among four superfamilies of Ensifera were (Grylloidea, (Tettigonioidea, (Hagloidea, Rhaphidophoroidea))).

## Introduction

Analyses of molecular data are often effective for phylogenetic reconstruction, as they benefit from a great number of informative characters without the kinds of biases that can be introduced by the choice and scoring of morphological characters [1]. The complete mitochondrial genome (mitogenome) is one of the most widely used molecular components in the phylogenetic analysis of insects, because it carries much more information than an individual gene.

The Ensifera consist of about 10,000 species and six extant superfamilies according to the Orthoptera Species File Online [2]. The group contains many familiar insects including katydids, crickets, mole crickets, and wetas. They are well known for acoustic signals produced in the contexts of courtship and mate recognition. Among the ensiferans are some clear relicts, such as members of Stenopelmatoidea and Hagloidea. The superfamily Hagloidea Handlirsch, 1906 was an ancient group of Ensifera. It was much more diverse in the past, extending at least into the Triassic, apparently diminishing in diversity through the Cretaceous [3]. It is the only family group of ensiferans, which can be traced from the Mesozoic to now [4]. Today, the Hagloidea are represented by only a few species, entirely in the sole modern family Prophalangopsidae Kirby, 1906 [2], that seems to intermingle traits of the Tettigonioidae and Grylloidea. The Prophalangopsidae contain five fossil subfamilies (Aboilinae Martynov, 1925; Protaboilinae Gorochov, 1988; Chifengiinae Hong, 1982; Termitidiinae Zeuner, 1939; Tettohaglinae Gorochov, 2003) and twoextant subfamilies: Prophalangopsinae Kirby, 1906 (extant 3 genera 4 species) and Cyphoderrinae Gorochov, 1988(extant 2 genera 4 species) [2].

The phylogeny of the Ensifera has been most investigated by Gwynne [5] and Desutter-Grandcolas [6]. However, the phylogenetic relationships between major ensiferan lineages are still poorly understood, despite many comprehensive efforts to define these lineages and reconstruct their relationships using morphological characters. The Hagloidea were an ancient group of Ensifera and appear to be ancestors of the Tettigonioidea and Stenopelmatoidea [7]. Prophalangopsidae is an intermediate group between Tettigonioidea, Stenopelmatoidea, and the more primitive Hagloidea. Therefore the above-mentioned trends are very important for their evolution (and consequently for their taxonomy) [7]. Prophalangopsinae and Cyphoderrinae are more closely related to the Tettigonioidae (katydids or bush-crickets) than to any other ensiferans, and their evolutionary split occurred more than 230 million years ago in the Permian. The close relationships between the Prophalangopsidae and Tettigonioidae are congruent with the results of molecular analysis [8].

Previously, the biology of the hump-winged cricket, *Tarragoilus diuturnus* Gorochov (Orthoptera: Hagloidea) from southwest China was mainly inferred based on the morphological and behavioural characters with which its taxonomic status was delineated. In this work, the complete mitogenome of T. diuturnus was sequenced. It is the first complete mitogenome from a representative of the ancient superfamily Hagloidea. The resultant data provide an opportunity to add this important group to the Ensifera phylogenetic analysis.

## Materials and Methods

### Ethics Statement

No specific permits were required for the insect collected for this study in Sichuan. The specimens of *T. diuturnus* were collected by using clap net at night. The field studies did not involve endangered or protected species. *T. diuturnus* was not included in the “List of Protected Animals in China”.

### Sample Origin and DNA Extraction

Specimens of *T. diuturnus* adult were collected from Liziping Nature Reserve (102.60°E, 29.09°N) in Sichuan Province, China, in July 2011. After collected, they were initially preserved in 100% ethanol in the field, and transferred to −20°C upon the arrival at the Hebei University.

Total DNA was extracted from the leg muscle tissue of a single adult male specimen using the TIANamp.

Genomic DNA Kit according to the manufacturer’s instructions (Tiangen Biotech, Beijing).

### Primer Design, PCR Amplification and Sequencing

First, three short fragments of *cox1*, *cox2* and *cytb* genes were determined respectively using the universal primer sets: LCO1490/HCO2198 for *cox1* [9], C2-J3399/TK-N3796 for *cox2* and CB-J10933/CB-N11367 for *cytb* [10], [11]. Based on this sequence information, two modified L-PCR primer sets [12] were used to amplify the entire *T. diuturnus* mitogenome into two overlapping larger fragments under the following conditions: a primary step denaturation at 94°C for 1 min, followed by 30 cycles of denaturation at 98°C for 10 s, annealing at 48°C for 30 s, and 68°C elongation for 10 min, followed by incubation at 68°C for 10 min. PCR reactions were performed in a MyCylerTM Personal Thermal Cycler (BIO-RAD) in 25 µL reaction volume set up as follows: 12.5 µL Premix LA Taq Hot Start (TaKaRa Biotech, Dalian), 1 µL of each primer (10 µM), 3 µL of genomic DNA, 7.5 µL of sterilized distilled water.

Sub-PCR amplifications were performed with L-PCR products as templates. In primer sets of Sub-PCR, one was designed based on the obtained sequence, and another was designed according to aligned mitogenome sequences of orthopterans in the NCBI nucleotide database and universal primers [10], [11], [13]. These primer pairs were then used within Sub-PCR amplifications to obtain adjacent gene sequences, which overlap with known sequences. In the same way, the cycle was repeated to ‘walk’ around the remainder of the genome. The Sub-PCR fragments were isolated and purified, and then ligated into PMD®-19 T vector (TaKaRa Biotech, Dalian) and sequenced from both strands on ABI 3730 XL DNA Analyzer (Biosune Biotech, Shanghai). All primer used for this study are included in Table S1.

### Genome Annotation, and Sequence Analysis

To prepare the mitogenome sequences for uploading into MOSAS, we first proofread and assembled raw sequence data into contigs using the Staden sequence analysis package [14], and then we uploaded entire mitogenome sequence files into MOSAS for annotation [15]. The nucleotide composition of different regions of the mitogenome and the codon usage of protein-coding genes were analyzed with MEGA 5.0 [16].

### Phylogenetic Analyses

A total of 17 taxa were analyzed in this study, including 15 ensiferan ingroup and two caeliferan outgroup taxa (Table S2). For this study, we sequenced 9 ensiferan species, *Ruspolia dubia* [17], *Gampsocleis gratiosa* [18], *Deracantha onos* [19], *Elimaea cheni* [20], *Conocephalus maculates* [21], *Xizicus fascipes* [22], *Mecopoda elongate* [23], *Mecopoda niponensis* [23] and *T. diuturnus*. Six additional ensiferan species were obtained from previous studies: *Anabrus simplex* [24], *Gryllotalpa orientalis* [25], *Gryllotalpa pluvialis* [26], *Myrmecophilus manni* [26], *Troglophilus neglectus* [26] and *Sinochlora longifissa* [27]. For outgroups we sampled two caeliferan species, *Locusta migratoria* (Caelifera: Acridomorpha) [28] and *Atractomorpha sinensis* (Caelifera: Pyrgomorphidae) [29].

DNA alignment of 13 protein-coding genes (PCGs) and two ribosomal (rRNA) genes was conducted using BioEdit [30] in which PCGs was inferred from the amino acid alignment. Alignments of individual genes were then concatenated excluding the stop codons. Maximum Likelihood (ML) using PhyML 3.0 [31], [32] and Bayesian inference analysis (BI) using MrBayes 3.1.2 [33] were used for phylogenetic reconstruction. In the ML method, a BioNJ tree was used as a starting tree to search for the ML tree with the GTR+I+Γ model. Robustness of the phylogenetic results was tested by bootstrap analysis with 1000 replicates. In BI analyses, ten million generations were run, with four MC chains, and trees were sampled every 1000 generations with a burnin of 25%. Trees inferred prior to stationarity were discarded as burnin, and the remaining trees were used to construct a 50% majority rule consensus tree.

## Results and Discussion

### Genome Organization

**Figure 1:**
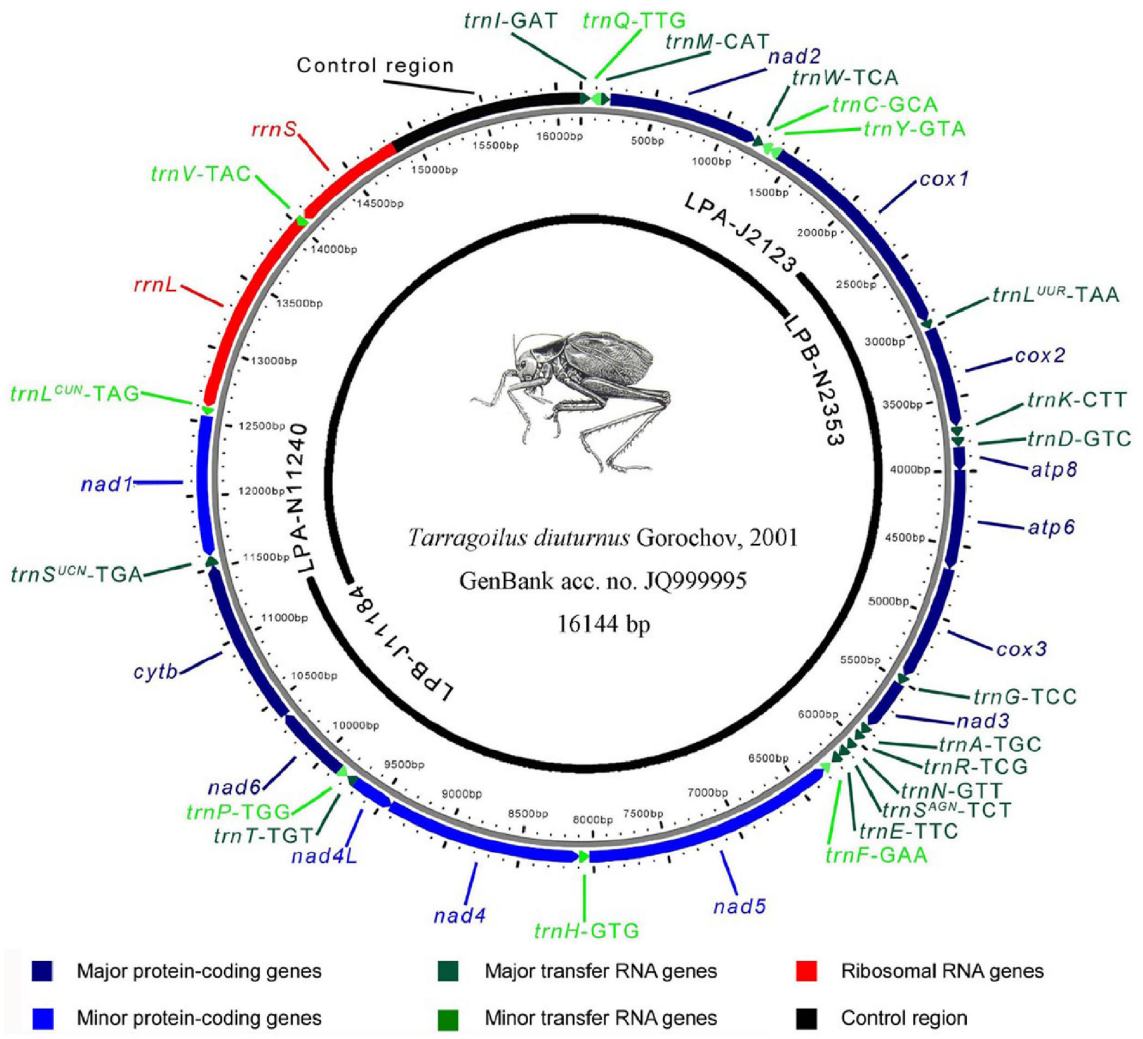
Mitochondrial genome of *Tarragoilus diuturnus*.

The *T. diuturnus* mitogenome is a typical circular DNA molecule of 16144 bp in length (GenBank accession number JQ999995; Figure 1). This length is medium-sized when compared with other orthopteran species, which typically range from 14,971 bp of *R. dubia* [17] to 18,133 bp of *S. longifissa* [27]. It has a typical gene content found in metazoan mitogenomes: 13 PCGs, 22 Transfer RNA (tRNA) genes, two rRNA genes, and one control region. The gene order and orientation of *T. diuturnus* mitogenome is identical to the hypothesized ancestral arthropod arrangement, found in *D. yakuba* and most ensiferans species [34]. Although the nucleotide composition of the *T. diuturnus* mitogenome is strongly biased towards A+T (67.21%), it is less so than most orthopterans (65.3%–76.2%, mean 73.2%) [18], [26].

Without considering the control region, a total of 65 bp of noncoding nucleotides were scattered among 9 intergenic regions varying from 1 to 25 bp. The largest intergenic region (25 bp) was present between *trnR* and *trnN*. Overlapping genes were also found in the *T. diuturnus* mitogenome, in which 52 bp were identified as overlapping sequences ranging from 1 to 8 bp in 14 regions. As has often been found across hexapod, there are several gene overlaps, such as the open reading frames (ORF) of *atp6/atp8* and *nad4L/nad4*, and each of which is overlapped by seven nucleotides. They are thought to be translated as a bicstron [35].

### Protein-coding Genes

The total length of all 13 PCGs was 11,250 bp, and the overall A+T content of *T. diuturnus* PCGs was 66.03%. The initiation and termination codons were identified using the ORFs finder and by comparison with the situation in other orthopterans. The initiation and termination codon assignments attempt to minimize gaps between gene regions while also minimizing gene overlap. Five of the 7 potential initiation codons (ATN and NTG) [36] were utilized in *T. diuturnus* mitochondrial protein-coding genes: ATT (*nad6*), ATA (*cox2*, *nad1*, *nad2,* and *nad3*), ATC (*cox1*), ATG (*atp6*, *cox3, cytb*, *nad4, nad4L, nad5*) and GTG (*atp8*) (Table 1). We allowed gene overlap between adjacent genes but not between protein-coding genes and tRNAs. When a full termination codon (TAA or TAG) caused an overlap between a protein-coding gene and a tRNA, we annotated this gene with incomplete termination codon T/TA instead (Table 1). We annotated full termination codons, however, in overlapping protein-coding genes. Six of the 13 protein-coding genes have incomplete termination codons T (*cox1*, *cox2*, *cytb*, *nad2*, *nad4*, and *nad5*), and six with termination codons TAA (*atp6*, *cox3*, *nad1*, *nad3*, *nad4L*, and *nad6*), one with TAG (*atp8*). The presence of incomplete termination codon is common in metazoan mitochondrial genomes, and polyadenylation following transcription likely converts these partial stop codons into full stop codons [37].

**Table 1:** Positions and nucleotide sequence lengths of mitochondrial genome of *Tarragoilus diuturnus*, and initiation and termination codons for protein-coding genes as well as tRNA gene anticodons (starting from *trnI*).

Gene/Region	Strand	Position	Size (bp)	Anticodon	Initiation/Termination	Intergenic nucleotides*
*trnI*	J	1–67	67	GAT		3
*trnQ*	N	71–139	69	TTG		−1
*trnM*	J	139–207	69	CAT		0
*nad2*	J	208–1234	1027		ATA-T	0
*trnW*	J	1235–1300	66	TCA		−8
*trnC*	N	1293–1358	66	GCA		5
*trnY*	N	1364–1428	65	GTA		−8
*cox1*	J	1421–2960	1540		ATC-T	0
*trnL^UUR^*	J	2961–3028	68	TAA		0
*cox2*	J	3029–3719	691		ATA-T	0
*trnK*	J	3720–3789	70	CTT		0
*trnD*	J	3790–3855	66	GTC		0
*atp8*	J	3856–4014	159		GTG-TAG	−7
*atp6*	J	4008–4685	678		ATG-TAA	−1
*cox3*	J	4685–5473	789		ATG-TAA	4
*trnG*	J	5478–5544	67	TCC		0
*nad3*	J	5545–5898	354		ATA-TAA	6
*trnA*	J	5905–5967	63	TGC		3
*trnR*	J	5971–6035	65	TCG		25
*trnN*	J	6061–6126	66	GTT		0
*trnS^AGN^*	J	6127–6193	67	GCT		3
*trnE*	J	6197–6261	65	TTC		−2
*trnF*	N	6260–6327	68	GAA		0
*nad5*	N	6328–8062	1735		ATG-T	0
*trnH*	N	8063–8125	63	GTG		0
*nad4*	N	8126–9464	1339		ATG-T	−7
*nad4L*	N	9458–9754	297		ATG-TAA	2
*trnT*	J	9757–9819	63	TGT		−1
*trnP*	N	9819–9888	70	TGG		1
*nad6*	J	9890–10417	528		ATT-TAA	−1
*cytb*	J	10417–11551	1135		ATG-T	0
*trnS^UCN^*	J	11552–11619	68	TGA		16
*nad1*	N	11636–12586	951		ATA-TAA	0
*trnL^CUN^*	N	12587–12650	64	TAG	0	
*rrnL*	N	12651–13990	1340		0	
*trnV*	N	13991–14060	70	TAC	0	
*rrnS*	N	14061–14843	783		0	
Control region		14844–16144	1301			

indicates gap nucleotides (Positive value) or overlapped nucleotides (Negative value) between two adjacent genes.

All codons are present in the protein-coding genes of this mitogenome. Excluding termination codons, there are a total of 3741 codons in the *T. diuturnus* protein-coding genes. The relative synonymous codon frequencies of *T. diuturnus* protein-coding genes are summarized in Table S3. The codon usage in *T. diuturnus* appears to be typical of other insect mitochondrial sequences. The synonymous codon usage shows a distinct bias towards T in the second and T/A in the third codon positions, regardless of the identity of the anti-codon encoded by the tRNA. In order, the four most used amino acids in *T. diuturnus* are Leu (15.64%), Ser (8.66%), Phe (8.55%), and Ile (8.29%).

### Transfer RNAs

**Figure 2:**
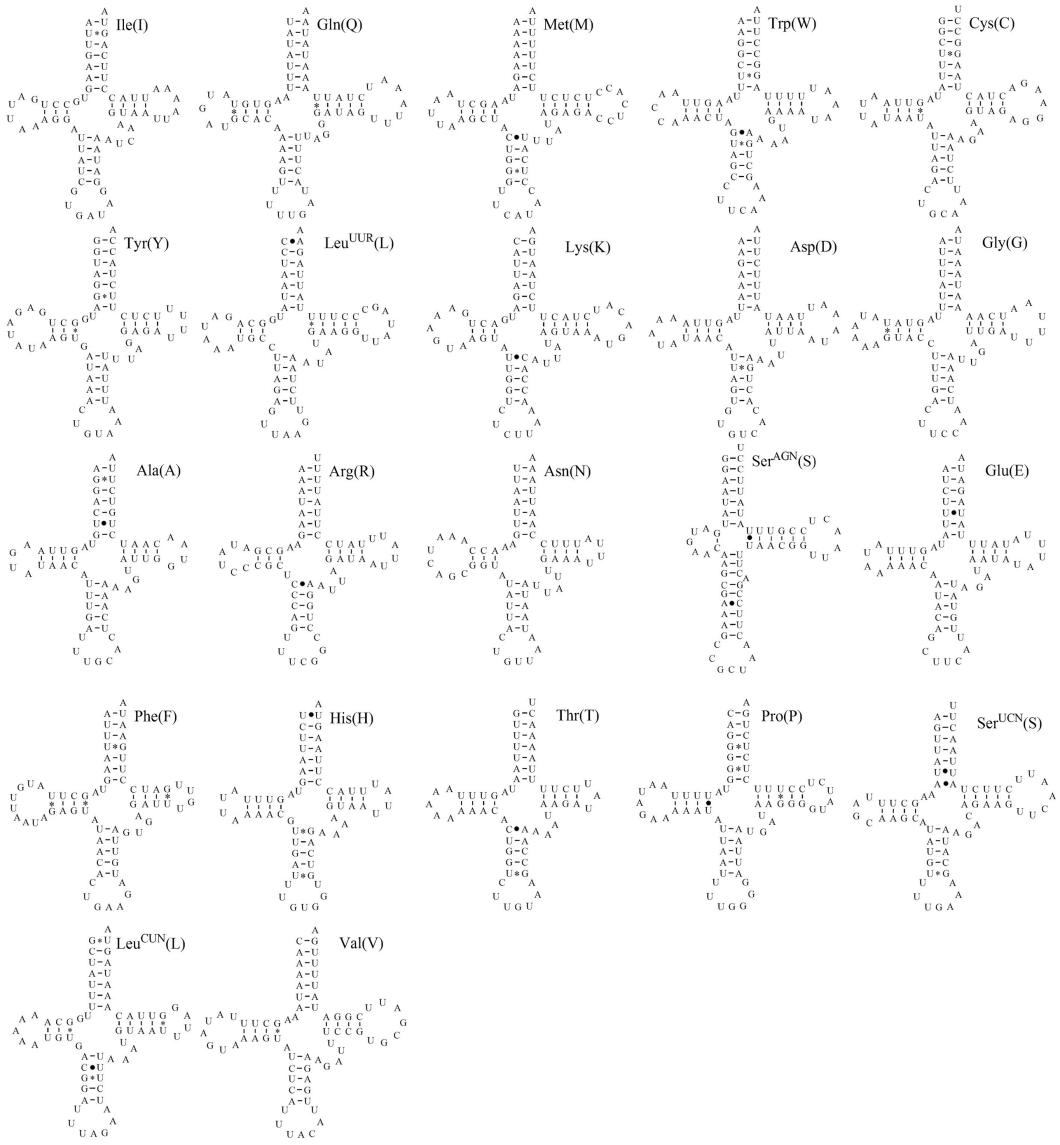
Putative secondary structures for 22*Tarragoilus diuturnus* mitochondrial genome. The tRNAs are labeled with abbreviations of their corresponding amino acids. Dashes (−) indicate Watson-Crick base-pairing, centered asterisks (*) indicate G-U base-pairing, and periods (•) indicate mismatches.

The complete set of 22 tRNA genes is present in the *T. diuturnus* mitogenome, and their length ranges from 63 to 70 bp. The relative locations for each tRNA gene are shown in Figure 1. All tRNA genes have the typical cloverleaf structures except for *trnS^AGN^*. The *trnS^AGN^* was found to have a lengthened anticodon stem (nine base-pairs) with a bulged nuleotide in the middle, an unusual T-stem (six base-pairs in constrast to the normal five base-pairs), a mini DHU arm (one base-pair) and no connector nucleotides (Figure 2) [38].

Thirty non-Watson-Crick pairings (G-U) and fifteen mismatches were identified in the *T. diuturnus* mitogenome tRNA genes. Six U/U were proposed in the acceptor stem of *trnH*, *trnS^UCN^*, *trnA*, *trnE*, the DHU stem of *trnP*, and the TψC stem of *trnS^AGN^*; three U/C were proposed in the anticodon stem of *trnL^CUN^*, *trnK* and *trnM*; four A–C in the anticodon stem of *trnT*, *trnR*, *trnSAGN*, and the acceptor stem of *trnLUUR*; one A-A was proposed in the acceptor stem of *trnS* ^UCN^ and one G-A in the anticodon stem of *trnW* (Figure 2).

### Ribosomal RNAs and Control Region

As in most insect mitogenomes, two rRNA genes (*rrnL* and *rrnS*) were present in the *T. diuturnus* mitogenome between *trnL^CUN^* and the control region, and separated by *trnV*. The lengths of *rrnL* and *rrnS* were respectively determined to be 1340 and 783 bp. The A+T content of *rrnL* and *rrnS* were 71.79% and 66.67%, respectively.

The 1301 bp *T. diuturnus* control region was observed in the conserved location between *rrnS* and *trnI*, and its composition was 66.18% A+T. The size of the *T. diuturnus* control region is well within the range found in other orthopterans, from 70 bp in *R. dubia* [17] to 2277 bp in *P. variolosa* [15]. The size differences of the control region occur not only because of its high rate of nucleotide substitution, insertion or deletion, but also due to the length of tandem repeat unit and the number of tandem repetitions.

### Phylogenetic Relationships

**Figure 3:**
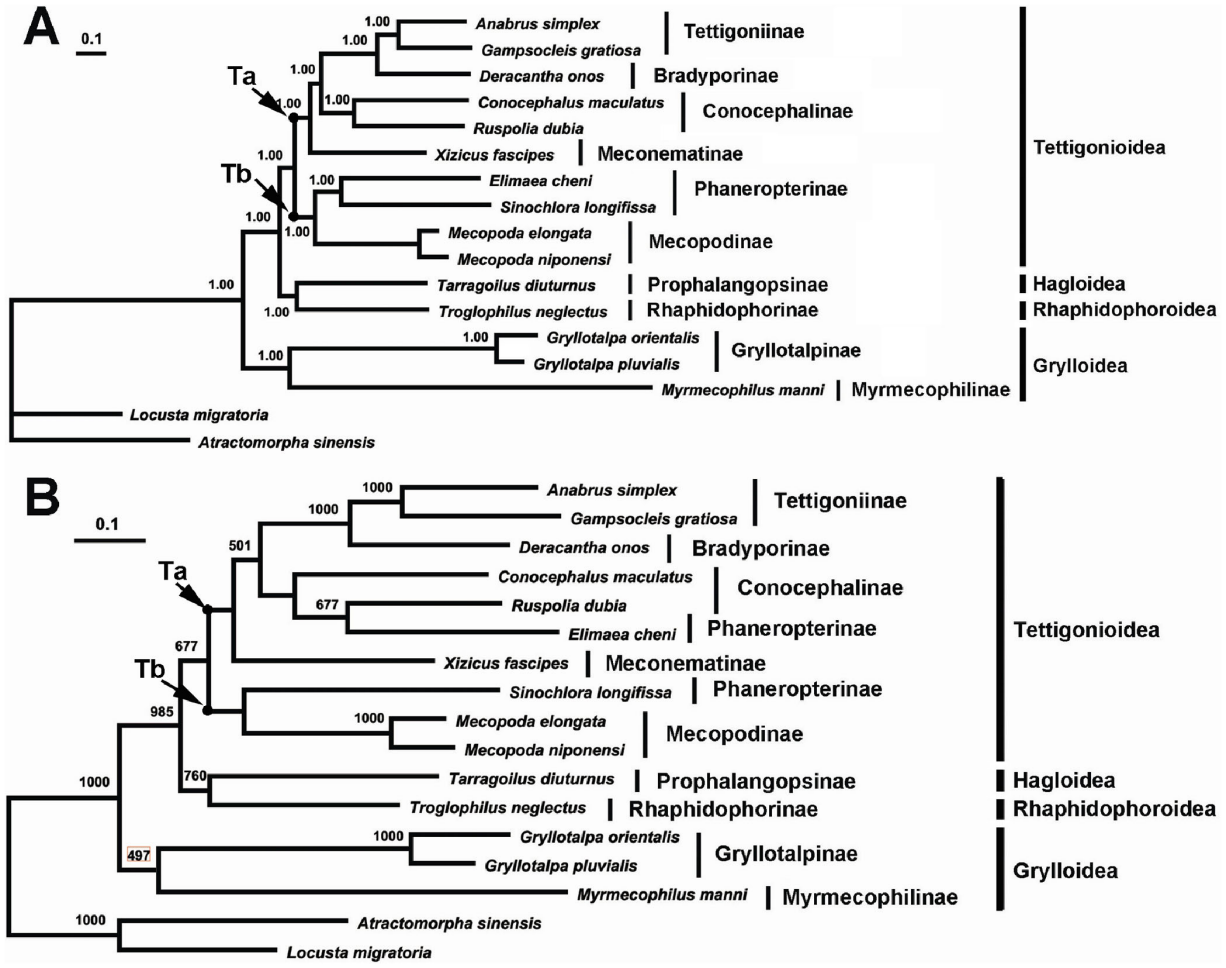
Phylogenetic reconstruction of the Ensifera using mitochondrial PCGs and rRNAs concatenated dataset. (A) Bayesian result, applicable posterior probability values are shown; (B) Maximum likelihood result with applicable bootstrap values (>50%) are shown.

The dataset (13,416 sites) used in the present analyses contained all three codon positions of 13 PCGs and two rRNA genes. The two independent runs yielded identical 50% consensus topologies, with harmonic means –ln = 142680.07 and 142679.11, respectively, and standard deviation of split frequencies <0.01 (Figure 3A). Hagloidea was placed as the sister group of Rhaphidophoroidea, and Tettigonioidea was a sister to Hagloidea+Rhaphidophoroidea. Both Tettigonioidea and Grylloidea were recovered as a monophyletic superfamily. Within Tettigonioidea, six subfamilies were divided into two large clades Ta (including Tettigoniinae, Bradyporinae, Conocephalinae and Meconematinae) and Tb (including Phaneropterinae and Mecopodinae). The best ML tree (Figure 3B) was found to have a score of Log-likelihood −146260.61551, and its topology was nearly identical to that recovered in the Bayesian analysis. The only exception was that the position of *E. cheni* (Phaneropterinae) shifted from clade Ta to Tb, and nested within Conocephalinae.

The Ensifera have usually been viewed as monophyletic group. Several attempts have been made to reconstruct the phylogeny of the Ensifera. Cranston and Gullan proposed Grylloidea was the sister group (but highly divergent, with a longer branch separation) of the remaining ensiferan taxa, including Tettigonioidea, Hagloidea, and Stenopelmatoidea [39]. Ander proposed dividing Ensifera into two major groups, which he called Grylloidea (including Gryllidaeand and Gryllotalpidae) and Tettigonioidea ((Rhaphidophoridae, (Schizodactylidae, (Gryllacrididae, (Stenopelmatidae, Prophalangopsidae, Tettigoniidae))))) [40].

Previous studies using morphological and molecular evidence have resulted in various hypotheses for the taxonomic status of the Hagloidea. Tettigoniidae and Hagloidae form a monophyletic group, sister to Stenopelmatidae and relatives (mormon crickets, wetas, cooloola monsters, and the like), but alternative analyses suggest different relationships, and conservatively, an unresolved group is perhaps appropriate at this stage [39]. Gorochov considered Prophalangopsidae (the sole extant family of Hagloidea) to be ancestral to the Tettigonioidae and Stenopelmatoidea [7]. Gwynne considered the Hagloidea to be a sister group of Tettigonioidae [5]. Jost and shaw considered the haglid Cyphoderris to be basal (or sister) to a clade of Grylloidea+Tettigoniidae (the families, Haglidae and Tettigoniidae used by authors) [1]. Flook *et al.* found the relationships among Tettigonioidae, Hagloidea and Stenopelmatoidea (including Stenopelmatidea, Schizodactylidea and Rhaphidophoridea, which were elevated to superfamily status in Orthoptera Species File Online) to be unresolved because different analyses gave conflicting results [8]. Mugleston *et al*. found the sister superfamily of Tettigonioidae still unresolved, it was Rhaphidophoroidae in the ML and BI analyses, whereas was Hagloidea in the MP analysis (the families, Tettigoniidae, Rhaphidophoridea and Prophalangopsidae used by authors) [41]. Regardless of the method of tree reconstruction used, the new mitochondrial data presented here strongly supports the Hagloidea *T. diuturnus* and Rhaphidophoroidea *T. neglectus* as forming a monophyletic group, sister to the Tettigonioidea. The relationships among the four superfamilies were (Grylloidea, (Tettigonioidea, (Hagloidea, Rhaphidophoroidea))).

## Supporting Information

Table S1 **Primer pairs used in PCR amplification of the** ***Tarragoilus diuturnus*** **mitogenome.**
(DOC)

Table S2 **Taxon samples, mitochondrial genome sequence accession numbers, and representative subfamilies following the classification of Otte (1997a,b, 2000).**
(DOC)

Table S3 **Relative synonymous codon usage of** ***Tarragoilus diuturnus*** **mitochondrial protein-coding genes is given in parantheses following the codon frequency.**
(DOC)
