# Correction: The First Mitochondrial Genome for the Superfamily Hagloidea and Implications for Its Systematic Status in Ensifera

**DOI:** 10.1371/journal.pone.0097542

**Published:** 2014-05-08

**Authors:** The PLOS ONE Staff

There is an error in Table 1. The last 4 data values of the “Intergenic nucleotides” column are incorrectly placed in the “Initiation/Termination” column. The authors have included a corrected version of the table here.

**Table 1:** **Positions and nucleotide sequence lengths of mitochondrial genome of *Tarragoilus diuturnus*, and initiation and termination codons for protein-coding genes as well as tRNA gene anticodons (starting from *trnI*).**

Gene/Region	Strand	Position	Size(bp)	Anticodon	Initiation/Termination	Intergenicnucleotides*
*trnI*	J	1-67	67	GAT		3
*trnQ*	N	71-139	69	TTG		-1
*trnM*	J	139-207	69	CAT		0
*nad2*	J	208-1234	1027		ATA-T	0
*trnW*	J	1235-1300	66	TCA		-8
*trnC*	N	1293-1358	66	GCA		5
*trnY*	N	1364-1428	65	GTA		-8
*cox1*	J	1421-2960	1540		ATC-T	0
*trnL^UUR^*	J	2961-3028	68	TAA		0
*cox2*	J	3029-3719	691		ATA-T	0
*trnK*	J	3720-3789	70	CTT		0
*trnD*	J	3790-3855	66	GTC		0
*atp8*	J	3856-4014	159		GTG-TAG	-7
*atp6*	J	4008-4685	678		ATG-TAA	-1
*cox3*	J	4685-5473	789		ATG-TAA	4
*trnG*	J	5478-5544	67	TCC		0
*nad3*	J	5545-5898	354		ATA-TAA	6
*trnA*	J	5905-5967	63	TGC		3
*trnR*	J	5971-6035	65	TCG		25
*trnN*	J	6061-6126	66	GTT		0
*trnS^AGN^*	J	6127-6193	67	GCT		3
*trnE*	J	6197-6261	65	TTC		-2
*trnF*	N	6260-6327	68	GAA		0
*nad5*	N	6328-8062	1735		ATG-T	0
*trnH*	N	8063-8125	63	GTG		0
*nad4*	N	8126-9464	1339		ATG-T	-7
*nad4L*	N	9458-9754	297		ATG-TAA	2
*trnT*	J	9757-9819	63	TGT		-1
*trnP*	N	9819-9888	70	TGG		1
*nad6*	J	9890-10417	528		ATT-TAA	-1
*cytb*	J	10417-11551	1135		ATG-T	0
*trnS^UCN^*	J	11552-11619	68	TGA		16
*nad1*	N	11636-12586	951		ATA-TAA	0
*trnL^CUN^*	N	12587-12650	64	TAG		0
*rrnL*	N	12651-13990	1340			0
*trnV*	N	13991-14060	70	TAC		0
*rrnS*	N	14061-14843	783			0
Control region		14844-16144	1301			

* indicates gap nucleotides (Positive value) or overlapped nucleotides (Negative value) between two adjacent genes.

## References

[1] Zhou Z, Shi F, Zhao L (2014) The First Mitochondrial Genome for the Superfamily Hagloidea and Implications for Its Systematic Status in Ensifera. PLoS ONE 9(1): e86027 doi:10.1371/journal.pone.0086027 24465850 10.1371/journal.pone.0086027 PMC3897600

