# Supplementary material for: The First Mitochondrial Genome for the Superfamily Hagloidea and Implications for Its Systematic Status in Ensifera
Source: PLoS One. 2014 Jan 21;9(1):e86027. doi: 10.1371/journal.pone.0086027 (PMC3897600; doi:10.1371/journal.pone.0086027)
Supplement: Table S1 — Primer pairs used in PCR amplification of the Tarragoilus diuturnus mitogenome. (DOC) [file pone.0086027.s001.doc]

Table 1. Primer pairs used in PCR amplification of the *Tarragoilus diuturnus* mitogenome

|  | Primer F | Sequence (5'→3') | Primer R | Sequence (5'→3') | Product size (kb) |
| --- | --- | --- | --- | --- | --- |
| Preliminary PCR | LCO-1490^b^ | TCAACAAATCATAAGGACATTGG | HCO-2198^b^ | TAAACTTCAGGGTGTCCAAAGAATCA | 0.7 |
|  | C2-J3399^a^ | ACAATTGGACAYCAATGATAYTG | TK-N3796^a^ | ACTATAAAATGGTTTAAGAGACC | 0.4 |
|  | CB-J10933^a^ | GTTTTACCATGAGGNCAAATRTC | CB-N11367^a^ | ATAACTCCTCCTAATTTATTAGGAAT | 0.4 |
| L-PCR | LPA-J2123^c,*^ | CACTTATTTTGATTTTTTGGTCACCCTGAAGT | LPA-N11240^c,*^ | AAGATAGCATAGGCAAATAGGAAGTATCACTC | 9 |
|  | LPB-J11184^c,*^ | AGTTACCCCCGTCCATATTCAACCTGAGTGAT | LPB-N3651^c,*^ | TGATTTGCCCCACAGATTTCGGAACATTGTCC | 7.5 |
| 1st round Sub-PCR | TW-J1301^a^ | GTTAANCAAACTAATARCYTTCAAA | HCO-2198^b^ | TAAACTTCAGGGTGACCAAAAAATCA | 0.9 |
|  | LPA-J2123^c,*^ | CACTTATTTTGATTTTTTGGTCACCCTGAAGT | LPB-N3651^c,*^ | TGATTTGCCCCACAGATTTCGGAACATTGTCC | 1.5 |
|  | CB-J10933^a^ | GTTTTACCATGAGGNCAAATRTC | LPA-N11240^c,*^ | AAGATAGCATAGGCAAATAGGAAGTATCACTC | 0.3 |
|  | LBP-J11184^c,*^ | AGTTACCCCCGTCCATATTCAACCTGAGTGAT | N1-N12242^a^ | GTTGCTCAAACTATTTCTTATGA | 1 |
| 2nd round Sub-PCR | B21-F32-36^d^ | TAAAGGATTAYYGTGATAG | N2-N1406^a.*^ | ATAAGTGATAAACTGTAAAT | 1.4 |
|  | A02-F03-3563^d,*^ | ATTGATGCCACCCCCGGCCG | A02-R04-4483^d^ | TAGGGTCCCTGGCCGAATTA | 0.9 |
|  | N4L-J9648^a^ | ACCCAAAGCACCCTCACAAAC | CB-N11010^a,*^ | TATCTACGGCGAATCCCCCTCA | 1.4 |
|  | B02-F10-11982^d,*^ | GGAGTTCGATTAGTTTCAGC | B02-R12-12845^d^ | GATTGCGACCTCGATGTTGG | 0.9 |
| 3rd round Sub-PCR | SR-J14610^a^ | ATAATMGGGTATCWAATCCTAGT | TM-N200^a,*^ | TCTCTATAAATGAGGTATGAACC | 1.7 |
|  | TK-J-3790 ^a,*^ | TCATCAGATGACTGAAAGTAAG | A6-N4552^a^ | ATGTCCWGCAATYATATTAGC | 0.8 |
|  | N4-J9172^a^ | CGTTCAGGTTGATAACCTCA | CB-N10608^a,*^ | CCTATATTTCATCATGCTGA | 1.4 |
|  | B03-F11-12755^d,*^ | GCTCACGCCGGTCTGAACTC | B03-R15-13290^d^ | CGCCTGTTTAACAAAAACAT | 0.5 |
| 4th round Sub-PCR | LR-J13900^a^ | ATAAACCCTGATACAMAAGGTAC | SR-N14745^a,*^ | GTGCCAGCTGCTGCGGTTAAAC | 0.8 |
|  | A6-J4463^a,*^ | CACCTAGTTCCTCAAGGAAC | C3-N5460^a^ | TCAACAAAGTGTCAGTATCA | 1.0 |
|  | A6-J4500^e,*^ | AACATAATCCGCCCAGGAAC | A16-R05-6062^d^ | CAGTAATATACCTCTYTTTGG | 1.5 |
|  | N4-J8641^a^ | GAWGAACATAAMCCATGACC | N4L-N9629^a,*^ | TGTGAGGGGGCCTTAGGATT | 1.0 |
| 5th round Sub-PCR | LR-J12888^a,*^ | CCGGTCTGAACTCAGATCATGTA | SR-N14220^a,*^ | ACATGCACATATCGCCCGTC | 1.3 |
|  | N5-J7572^a^ | GGAATTTGTGCTCTCTTWGT | N4-N8722^a,*^ | ATTGCTTATTCATCTGTTG | 1.2 |
|  | N3-J5762^e,*^ | TTTGACCCCAAGAGTTCCGC | R06-7061^d^ | AAGGATTCTCAGGATATTCG | 1.3 |
| 6th round Sub-PCR | TF-J6400^a,*^ | TAACATCTTCAGTGTTATACTCT | N5-N7793^a,*^ | TTAGGTTGGGATGGGTTGGG | 1.4 |

^a^ Modified from Simon *et al.* (2006). ^b^ Modified from Folmer *et al.* (1994). ^c^ Modified from Liu *et al.* (2006). ^d^ Modified from Zhang and Huang (2008).^*^ Modified taxon-specific primers, while the others are Orthoptera-specific.
