# Supplementary material for: The First Mitochondrial Genome for the Superfamily Hagloidea and Implications for Its Systematic Status in Ensifera
Source: PLoS One. 2014 Jan 21;9(1):e86027. doi: 10.1371/journal.pone.0086027 (PMC3897600; doi:10.1371/journal.pone.0086027)
Supplement: Table S2 — Taxon samples, mitochondrial genome sequence accession numbers, and representative subfamilies following the classification of Otte (1997a,b, 2000). (DOC) [file pone.0086027.s002.doc]

Table S2. Taxon samples, mitochondrial genome sequence accession numbers, and representative subfamilies following the classification of Otte (1997a,b, 2000)

| **Classification** | **Taxon** | **Accession number** |
| --- | --- | --- |
| Grylloidea |  |  |
| Myrmecophilidae: Myrmecophilinae | *Myrmecophilus manni* | EU938370 |
| Gryllotalpidae: Gryllotalpinae | *Gryllotalpa pluvialis* | EU938371 |
| Gryllotalpidae: Gryllotalpinae | *Gryllotalpa orientalis* | AY660929 |
| Tettigonioidea |  |  |
| Tettigoniidae: Tettigoniinae | *Gampsocleis gratiosa* | EU527333 |
| Tettigoniidae: Tettigoniinae | *Anabrus simplex* | EF373911 |
| Tettigoniidae: Bradyporinae | *Deracantha onos* | EU137664 |
| Tettigoniidae: Conocephalinae | *Ruspolia dubia* | EF583824 |
| Tettigoniidae: Conocephalinae | *Conocephalus maculates* | HQ711931 |
| Tettigoniidae: Phaneropterinae | *Elimaea cheni* | GU323362 |
| Tettigoniidae: Phaneropterinae | *Sinochlora longifissa* | KC467055 |
| Tettigoniidae: Meconematinae | *Xizicus fascipes* | JQ326212 |
| Tettigoniidae: Mecopodinae | *Mecopoda elongata* | JQ917910 |
| Tettigoniidae: Mecopodinae | *Mecopoda niponensis* | JQ917909 |
| Rhaphidophoroidea |  |  |
| Rhaphidophoridae: Rhaphidophorinae | *Troglophilus neglectus* | EU938374 |
| Hagloidea |  |  |
| Haglidae | *Tarragoilus diuturnus* | JQ999995 |
| Caeliferan outgroups |  |  |
| Acrididae | *Locusta migratoria* | X80245 |
| Pyrgomorphidae | *Atractomorpha sinensis* | EU263919 |
