# Supplementary material for: The First Mitochondrial Genome for the Superfamily Hagloidea and Implications for Its Systematic Status in Ensifera
Source: PLoS One. 2014 Jan 21;9(1):e86027. doi: 10.1371/journal.pone.0086027 (PMC3897600; doi:10.1371/journal.pone.0086027)
Supplement: Table S3 — Relative synonymous codon usage of Tarragoilus diuturnus mitochondrial protein-coding genes is given in parantheses following the codon frequency. (DOC) [file pone.0086027.s003.doc]

Table S3. Relative synonymous codon usage of *Tarragoilus diuturnus* mitochondrial protein-coding genes is given in parantheses following the codon frequency

| Codon | n | RSCU | Codon | n | RSCU | Codon | n | RSCU | Codon | n | RSCU |
| --- | --- | --- | --- | --- | --- | --- | --- | --- | --- | --- | --- |
| UUU(F) | 243 | 1.52 | UCU(S) | 84 | 2.07 | UAU(Y) | 112 | 1.46 | UGU(C) | 36 | 1.53 |
| UUC(F) | 77 | 0.48 | UCC(S) | 38 | 0.94 | UAC(Y) | 41 | 0.54 | UGC(C) | 11 | 0.47 |
| UUA(L) | 310 | 3.18 | UCA(S) | 74 | 1.83 | UAA(*) | 0 | 0 | UGA(W) | 79 | 1.48 |
| UUG(L) | 74 | 0.76 | UCG(S) | 10 | 0.25 | UAG(*) | 0 | 0 | UGG(W) | 28 | 0.52 |
| CUU(L) | 64 | 0.66 | CCU(P) | 69 | 1.79 | CAU(H) | 50 | 1.22 | CGU(R) | 10 | 0.67 |
| CUC(L) | 33 | 0.34 | CCC(P) | 41 | 1.06 | CAC(H) | 32 | 0.78 | CGC(R) | 9 | 0.6 |
| CUA(L) | 89 | 0.91 | CCA(P) | 39 | 1.01 | CAA(Q) | 60 | 1.48 | CGA(R) | 26 | 1.73 |
| CUG(L) | 15 | 0.15 | CCG(P) | 5 | 0.13 | CAG(Q) | 21 | 0.52 | CGG(R) | 15 | 1 |
| AUU(I) | 218 | 1.41 | ACU(T) | 80 | 1.35 | AAU(N) | 105 | 1.43 | AGU(S) | 45 | 1.11 |
| AUC(I) | 92 | 0.59 | ACC(T) | 58 | 0.98 | AAC(N) | 42 | 0.57 | AGC(S) | 15 | 0.37 |
| AUA(M) | 195 | 1.67 | ACA(T) | 93 | 1.57 | AAA(K) | 52 | 1.32 | AGA(S) | 55 | 1.36 |
| AUG(M) | 39 | 0.33 | ACG(T) | 6 | 0.1 | AAG(K) | 27 | 0.68 | AGG(S) | 3 | 0.07 |
| GUU(V) | 54 | 1.01 | GCU(A) | 76 | 1.42 | GAU(D) | 51 | 1.32 | GGU(G) | 44 | 0.74 |
| GUC(V) | 28 | 0.52 | GCC(A) | 63 | 1.18 | GAC(D) | 26 | 0.68 | GGC(G) | 21 | 0.35 |
| GUA(V) | 93 | 1.74 | GCA(A) | 64 | 1.2 | GAA(E) | 51 | 1.32 | GGA(G) | 80 | 1.34 |
| GUG(V) | 39 | 0.73 | GCG(A) | 11 | 0.21 | GAG(E) | 26 | 0.68 | GGG(G) | 94 | 1.57 |
